# Isolated pterygium colli in a child: case report and literature review of surgical techniques

**DOI:** 10.1097/RC9.0000000000000463

**Published:** 2026-04-23

**Authors:** Badr Saout Arrih, Walid Bijou, Sami Rouadi, Reda Abada, Mohamed Mahtar

**Affiliations:** Department of Otolaryngology, Head and Neck Surgery, Ibn Rochd University Hospital, Faculty of Medicine and Pharmacy, Hassan II University, Casablanca, Morocco

**Keywords:** congenital malformation, pediatric surgery, pterygium colli, surgical techniques, webbed neck

## Abstract

**Background and importance:**

Pterygium colli or “webbed neck” constitutes a rare congenital malformation characterized by bilateral cutaneous excess extending from the mastoid region to the acromion. Although of low incidence in the general population, this anomaly can lead to significant functional limitations and considerable psychosocial impact, particularly in children and adolescents.

**Case presentation:**

We report the case of an 8-year-old girl with no particular medical history, presenting bilateral cervical webbing of cutaneous appearance giving a webbed neck appearance, present since birth. Preoperative genetic screening confirmed a normal 46, XX karyotype, and clinical evaluation ruled out Noonan and Klippel–Feil syndromes. Objective measurements revealed restricted cervical rotation (45°) and lateral flexion (25°). Clinical examination revealed bilateral malformation involving exclusively the cutaneous plane, extending from the mastoid to the acromion, without associated inflammatory signs. Surgical management by bilateral excision of an ellipsoidal portion of skin with two-layer closure using unilateral advancement flaps was performed. The postoperative course was uncomplicated with discharge on postoperative Day 1.

**Clinical discussion:**

Recent literature review highlights significant evolution in surgical techniques for pterygium colli. Therapeutic approaches diversify between lateral techniques and posterior approaches. Turki’s classification distinguishes three morphological types guiding optimal technical choice. Severe forms may require staged surgical approach combining multiple techniques. Our 12-month follow-up demonstrates sustained outcomes, with range of motion returning to normal physiological values (rotation 80°, lateral flexion 40°).

**Conclusion:**

Isolated pterygium colli in children constitutes a justified surgical indication due to its functional and aesthetic repercussions. The development of less invasive techniques and optimization of protocols allows for individualized approach, according to specific morphological characteristics of each patient.

## Introduction

Pterygium colli, commonly called “webbed neck,” represents a rare congenital malformation characterized by the presence of bilateral cutaneous excess extending from the mastoid region to the acromion. This developmental anomaly may present in isolated form or be associated with various syndromic pathologies, notably Turner syndrome, Noonan syndrome, or Klippel–Feil syndrome^[^1^]^.

Given these strong syndromic associations, a systematic etiological evaluation is imperative before any surgical intervention. Turner syndrome affects approximately 1 in 2500 female births and presents with pterygium colli in 75% of cases. Similarly, Noonan syndrome (1 in 1000–2500 births) and Klippel–Feil syndrome require specific screening to detect associated cardiac, renal, or skeletal anomalies that could influence anesthetic management and surgical planning^[^2^]^.

Although this malformation presents relatively low incidence in the general population, its functional and psychosocial impact can be considerable, particularly in children and adolescents. Limitations of cervical mobility, difficulties in daily activities, and aesthetic repercussions justify appropriate surgical management^[^1,3^]^.

The evolution of surgical techniques over the last decade has significantly improved aesthetic and functional results. Therapeutic approaches have diversified, ranging from traditional lateral techniques using Z-plasty and its variants to more recent posterior approaches such as “posterior cervical lift,” offering the advantage of better scar concealment^[^4–9^]^.

The recent classification proposed by Turki and collaborators in 2024 provides an updated conceptual framework allowing guidance of optimal surgical technique choice based on specific morphological characteristics of each patient^[^1^]^.

The objective of this paper is to report a case of isolated pterygium colli in an 8-year-old girl treated by bilateral excision with closure by advancement flaps and to present an updated literature review concerning different available surgical techniques and their results.

This case report has been reported in line with the SCARE criteria^[^10^]^.

HIGHLIGHTS

- Excellent pediatric surgical outcomes demonstrated in an 8-year-old patient with isolated pterygium colli, achieving complete functional restoration and optimal aesthetic results through bilateral excision with unilateral advancement flaps.
- Early discharge protocol successfully implemented with day-1 postoperative discharge, combined with rigorous long-term monitoring, demonstrating both the safety and effectiveness of minimally invasive approaches in pediatric webbed neck correction.
- Pediatric-specific surgical considerations including age-appropriate anesthetic management, tissue handling techniques, and postoperative care protocols optimized for younger patients with isolated pterygium colli.
- Twelve-month follow-up results showing complete healing without complications, absence of infection, excellent scar maturation, and full range of cervical motion restoration in pediatric patient confirmed by objective goniometric measurements.
- Validation of advancement flap technique as the optimal age-appropriate surgical approach for isolated pediatric cases (Turki Type I), offering superior outcomes with minimal invasiveness and reduced operative time compared to complex reconstructive procedures.

## Case report

We report the case of an 8-year-old girl with no particular medical history, admitted to our ENT department for bilateral cervical webbing of cutaneous appearance giving a webbed neck aspect, present since birth. The patient is afebrile and in good general condition (Table 1).

**Table 1:** Timeline.

Timepoint	Event
Birth	Parents noted webbed appearance of the neck. No other congenital anomalies detected.
Age 8 years (Month 0)	Presentation to ENT department. Clinical diagnosis of pterygium colli. Syndromic workup initiated.
Month 1	Completion of genetic and orthopedic evaluation. Diagnosis of “Isolated Pterygium Colli” confirmed. Preoperative planning.
Month 2 (Day 0)	Surgical intervention: Bilateral ellipsoidal excision with unilateral advancement flaps.
Post-op Day 1	Discharge from hospital.
Post-op Day 7	Suture removal. Wound check: clean healing.
Post-op Months 1, 3, 6	Follow-up visits: monitoring scar maturation and mobility.
Post-op Month 12	Final evaluation: assessment of range of motion, aesthetic outcome, and scar quality.

A comprehensive syndromic evaluation was conducted prior to surgical planning. Genetic evaluation revealed a normal karyotype 46, XX, excluding Turner syndrome. Clinical examination showed no features of Noonan syndrome (normal cardiac evaluation, normal developmental milestones) or Klippel–Feil syndrome (normal cervical spine X-ray, full vertebral mobility) (Table 1).

On general physical examination, the patient is in good hemodynamic, respiratory, and neurological condition (Table 1).

**Figure 1.**
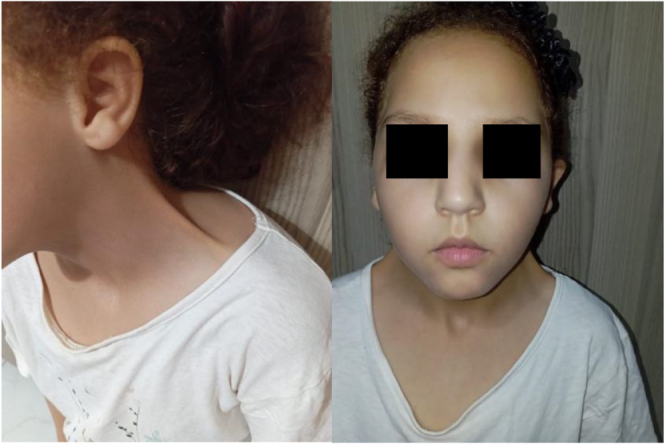
Photograph of the child showing the pterygium colli appearance.

On cervical examination, we note a bilateral malformation involving the cutaneous plane alone, giving the neck a webbed appearance, extending from the mastoid to the acromion without inflammatory signs. A decrease in neck mobility in rotation and anteflexion was also noted (Fig. 1).

Preoperative objective functional assessment was performed using a universal goniometer. Measurements revealed cervical rotation limited to 45° bilaterally (normal pediatric range: 80–90°) and lateral flexion limited to 25° (normal pediatric range: 40–45°). The skinfold thickness was measured at 2.5 cm at the mid-cervical level.

The rest of the ENT examination (otoscopy, rhinoscopy, and oral cavity) was normal.

Given this clinical presentation, and the confirmation of an isolated Type I deformity (Turki classification), the decision for surgical treatment was made (Table 1).

**Figure 2.**
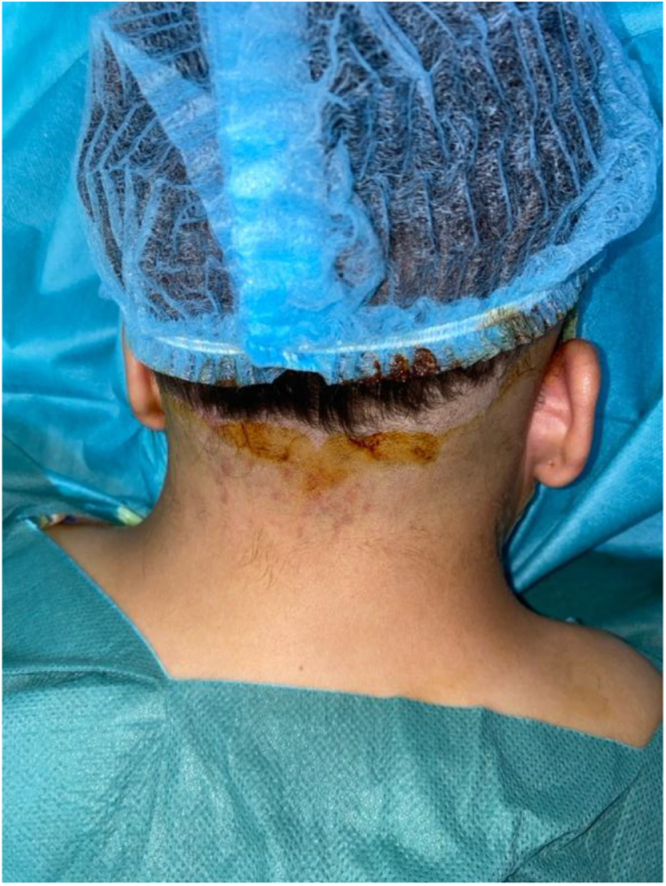
Surgical position in prone decubitus of the patient.

**Figure 3.**
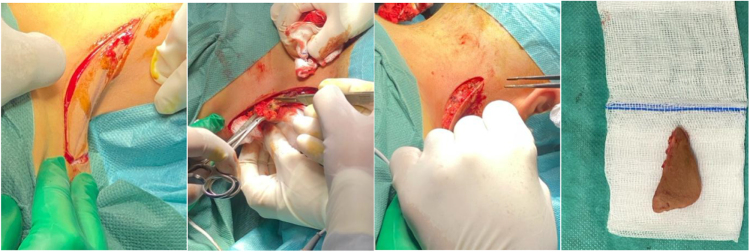
Bilateral excision of an ellipsoidal portion of skin.

**Figure 4.**
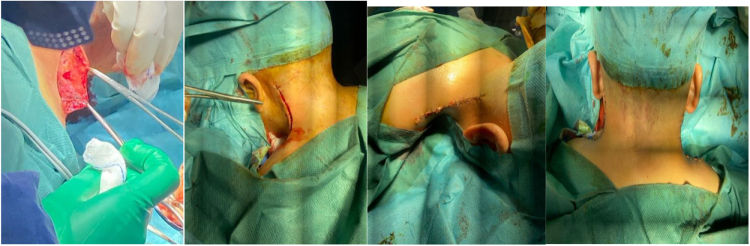
Two-layer closure subcutaneous and cutaneous using unilateral advancement flaps.

Surgery was performed by an ENT surgeon with 15 years of experience in cervicofacial reconstructive surgery, with the patient in prone position (Fig. 2). It consisted of bilateral excision of an ellipsoidal portion of skin and two-layer closure (subcutaneous and cutaneous) using unilateral advancement flaps (Figs 3 and 4). This technique was selected over Z-plasty to avoid the stigma of visible lateral scars and over the posterior lift due to the localized nature of the redundancy.

In the immediate postoperative period, the patient was placed on antibiotics (amoxicillin–clavulanic acid) and analgesics. The patient was discharged from the department on postoperative Day 1 (Table 1).

**Figure 5.**
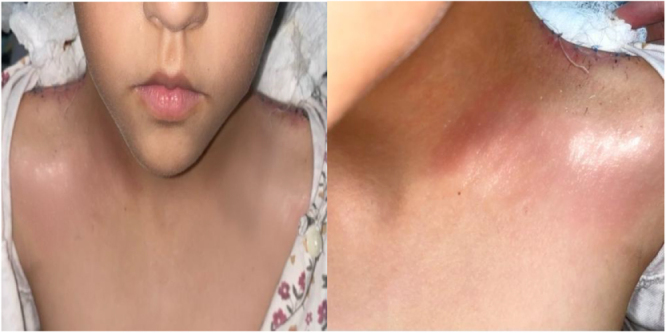
Seven days of follow-up, good healing, in terms of aesthetic appearance and neck mobility.

The follow-up was at 7 days postoperative. We noted excellent healing, in terms of aesthetic appearance and neck mobility (Fig. 5, Table 1).

Follow-up evaluations were subsequently conducted at 1, 3, 6, and 12 months postoperatively. At the 12-month follow-up, the scars were mature, flat, and well-concealed within the natural skin tension lines. There was no evidence of hypertrophic scarring or keloid formation (Table 1).

Objective functional outcome measures at 12 months showed full restoration of cervical mobility: rotation improved to 80° bilaterally (from 45°) and lateral flexion improved to 40° (from 25°). No recurrence of the web was observed (Table 1).

The patient and her parents expressed high satisfaction with the procedure. The parents reported a significant improvement in the child’s self-esteem and social interaction at school. Specifically, the child noted she could now wear her hair up without embarrassment and participate in sports that required head turning without restriction. On a Visual Analog Scale for satisfaction (0–10), the parents rated the cosmetic outcome as 9/10 and functional improvement as 10/10 (Table 1).

## Discussion

Pterygium colli, also designated by the term “webbed neck,” constitutes a rare congenital malformation characterized by the presence of bilateral cutaneous excess extending from the mastoid region to the acromion^[^1^]^.

Our case reinforces the need for a systematic approach to diagnosis. While the association with Turner syndrome (45, X) is well-known, occurring in 20% of cases^[^11^]^, recent data emphasizes the overlap with Noonan syndrome, which may present with similar cervical features but distinct cardiac risks (pulmonary stenosis vs. coarctation of the aorta in Turner syndrome)^[^12^]^.

The SCARE 2025 guidelines emphasize the importance of negative findings in case reporting; identifying this case as “isolated” required rigorous exclusion of these syndromes.

The choice of bilateral ellipsoidal excision with unilateral advancement flaps for this patient was driven by the Turki Type I morphology (simple cutaneous excess without deep fibrous bands). Unlike Z-plasties, which effectively lengthen the scar but leave visible zigzag marks on the lateral neck^[^4^]^, or the posterior cervical lift, which is excellent for posterior redundancy but carries a higher risk of hypertrophic scarring^[^6,13^]^, our chosen technique balances scar concealment with effective contouring. The lateral location of the incision was placed to fall into natural neck creases as the child grows (Table 2).

**Table 2:** Comparison of modern surgical approaches for pterygium colli.

Technique	Best indication (Turki Class)	Recurrence rate	Complication profile	Scar visibility
Z-plasty & modifications	Type II (Fibrous bands)	Low (<10%)	Tip necrosis, trapdoor effect	High (Lateral neck)
Posterior cervical lift	Types I & III (Excess skin)	Low to Moderate	Hypertrophic scarring (15–20%)^[^9^]^	Low (Posterior midline/hairline)
Lateral advancement flaps (our case)	Type I (Isolated skin)	Low	Wound dehiscence (rare)	Moderate (Lateral, can be hidden)

A critical review of the recent literature reveals a shift toward minimizing visible scarring while maximizing functional gain^[^1,3–8,11,14–17^]^ (Table 2).

Recent studies by San Basilio *et al* suggest that for severe forms, a single approach is often insufficient, recommending a staged posterior and lateral approach^[^8^]^. However, for isolated cases like ours, the single-stage advancement flap proved sufficient. This contrasts with Jungbauer’s 2022 review, which advocated for Z-plasty as the gold standard; our findings align more with recent trends favoring aesthetic flap reconstruction^[^7^]^ (Table 2).

This malformation can lead to significant functional limitations, notably restriction of cervical mobility, difficulties during certain daily activities, and considerable psychosocial impact, particularly in children and adolescents^[^4–19^]^ (Table 2).

Pterygium colli presents relatively low incidence in the general population, but its prevalence increases significantly in association with certain genetic syndromes. Turner syndrome represents the most frequent association, with webbed neck prevalence observed in approximately 20% of patients affected by this chromosomal pathology. This association is particularly significant because the presence of pterygium colli in female newborns may constitute an early diagnostic sign of Turner syndrome^[^11^]^.

Noonan syndrome constitutes the second most frequently reported syndromic association in recent literature. Patients affected by this autosomal dominant syndrome often present cervical malformations similar to those observed in Turner syndrome, although clinical presentation may differ slightly. Klippel–Feil syndrome represents a third significant association, characterized by congenital fusion of cervical vertebrae frequently accompanied by pterygium colli. Isolated forms of pterygium colli, without evident syndromic association, have also been reported in recent literature^[^15,16,20^]^.

The physiopathology of pterygium colli remains partially elucidated, although several theories have been proposed. The most widely accepted hypothesis suggests embryonic origin linked to early fetal lymphedema, leading to accumulation of cutaneous and subcutaneous tissue in the cervical region^[^17^]^.

Turki and collaborators proposed in 2024 an updated classification of pterygium colli based on structural variations of the cutaneous fold. This classification distinguishes three main types: Type I characterized by simple cutaneous excess without underlying fibrous band (pseudo-webbing), Type II presenting significant subdermic fibrous band, and Type III combining major cutaneous excess with deep musculo-aponeurotic anomalies. This classification proves particularly useful for guiding optimal surgical technique choice^[^1^]^.

Lateral approach techniques historically constitute reference methods for pterygium colli correction. Z-plasty and its modified variants represent the most anciently described and most widely used techniques. The main advantage of these approaches lies in their capacity to effectively redistribute cutaneous tensions while allowing direct visualization of deep structures, thus facilitating excision of eventual fibrous bands^[^4^]^.

Reichenberger’s technique, developed and perfected over recent decades, proposes a sophisticated lateral approach using cervical advancement flap. This method allows effective correction of cervical contour with relatively low recurrence rate. However, it presents the disadvantage of creating visible scars on the anterolateral face of the neck^[^5^]^.

Mehri Turki’s technique, recently described and analyzed in a 2024 comparative study, represents significant evolution of lateral approaches. This technique uses modified cervical advancement flap allowing better scar concealment while maintaining optimal corrective efficacy. Turki’s comparative study classifies this technique in group 2 of his classification system, corresponding to techniques offering the best aesthetic results^[^1^]^ (Table 2).

The “posterior cervical lift,” initially described by Chaput and collaborators in 2013, revolutionized the surgical approach to pterygium colli. This technique presents the major advantage of concealing scars in the posterior capillary region, thus creating superior aesthetic result. The principle is based on excision of posterior cutaneous excess followed by advancement and fixation of superficial cervical fascia to the nuchal ligament^[^6^]^ (Table 2).

Recent modifications of this technique, reported in 2025, include the use of transfixion sutures of deep fascia with anchoring between the two cervical flaps, as well as personalized excision of redundant tissue at the distal aspect of the wound with local tissue rearrangement. These modifications aim to optimize tension distribution and reduce the risk of hypertrophic scarring^[^7^]^ (Table 2).

Severe forms of pterygium colli sometimes require staged surgical approach combining multiple techniques. San Basilio and collaborators recently reported the use of double approach, posterior and lateral, for correction of particularly important forms. This strategy allows separate treatment of different components of deformation while minimizing risks of complications related to extensive single intervention^[^2,8–10,12,13,18,19^]^.

Analysis of recent literature reveals variable success rates according to employed technique. Modified posterior approaches present aesthetic satisfaction rates superior to 85%. However, these techniques are associated with hypertrophic scarring risk, reported in 15–20% of cases according to recent series^[^2,9,10,12,13,18^]^.

Traditional lateral approaches show recurrence rates inferior to 10% but present the disadvantage of visible scars on the lateral face of the neck^[^1^]^.

Operating on an 8-year-old presents specific challenges regarding scar maturation and growth. Pediatric skin has higher tension and a greater propensity for hypertrophic scarring compared to adults. Our strict 12-month follow-up protocol was essential to monitor this risk. The decision to operate at age 8 allowed for cooperation with postoperative physical therapy to maintain range of motion (ROM), a crucial factor in the reported functional success (80° rotation)^[^21,22^]^.

A limitation of this report is the single-case nature. While the 12-month follow-up provides better evidence than immediate results, long-term follow-up into late adolescence would be ideal to assess the scar’s behavior during the pubertal growth spurt.

## Conclusion

Isolated pterygium colli in children constitutes a justified surgical indication due to its functional and aesthetic repercussions. Our 12-month outcomes validate the use of bilateral ellipsoidal excision with advancement flaps for Type I deformities, providing excellent ROM recovery and patient satisfaction. The development of less invasive techniques and optimization of protocols allows for individualized approach according to specific morphological characteristics of each patient.

## Data Availability

The data supporting the findings of this study are not publicly available due to patient privacy and confidentiality restrictions but are available from the corresponding author upon reasonable request.
